# Clinical Outcomes of Catheter Ablation for Atrial Fibrillation in Patients with Acute Decompensated Heart Failure

**DOI:** 10.3390/jcm14020629

**Published:** 2025-01-19

**Authors:** Yoshifumi Ikeda, Ritsushi Kato, Hitoshi Mori, Kenta Tsutsui, Kazuhisa Matsumoto, Masataka Narita, Wataru Sasaki, Daisuke Kudo, Naomichi Tanaka, Kazuo Matsumoto

**Affiliations:** Department of Cardiology, Saitama Medical University International Medical Center, Saitama 350-1298, Japan; ritsn@saitama-med.ac.jp (R.K.); zin_ndmc@yahoo.co.jp (H.M.); tsutsuik@saitama-med.ac.jp (K.T.); m_kazu0@saitama-med.ac.jp (K.M.); n_masa19@saitama-med.ac.jp (M.N.); wata1107@saitama-med.ac.jp (W.S.); dice-k.0506@outlook.jp (D.K.); ntanaka@saitama-med.ac.jp (N.T.); kazuom0225@hotmail.co.jp (K.M.)

**Keywords:** atrial fibrillation, catheter ablation, acute decompensated heart failure, heart failure

## Abstract

**Background**: The prognosis of acute decompensated heart failure (ADHF) and heart failure (HF) with atrial fibrillation (AF) has been dismal. This study was performed to investigate the clinical outcomes of catheter ablation (CA) performed in patients with concurrent ADHF and AF. **Methods**: We retrospectively analyzed ADHF patients with AF who were admitted to our institution from 2007 to 2017. **Results**: In total, 472 patients were included in this study, with a mean follow-up duration of 32.8 ± 32.9 months. The 5-year event-free rate (cardiovascular death and HF hospitalization) was 61.4%, and the 10-year event-free rate was 42.7%. A comparative analysis of the event group and control group revealed that patients in the event group were older (event group vs. control group: 72.1 ± 11.0 vs. 68.8 ± 13.4 years, *p* = 0.008) and had a higher proportion of Clinical Scenario 3 classifications (event group vs. control group: 24% vs. 12%, *p* = 0.001). Notably, patients in the event group had a lower sinus rhythm maintenance rate (event group vs. control group: 17% vs. 31%, *p* < 0.001) and CA rate (event group vs. control group: 9% vs. 21%, *p* = 0.003). The CA group had a higher event-free rate than the non-CA group, and this trend persisted even after matching the patients’ backgrounds (log-rank test: *p* < 0.001). **Conclusions**: Patients presenting with AF at the onset of ADHF showed a poor prognosis, whereas CA demonstrated potential for improving the prognosis for some of these patients.

## 1. Introduction

Atrial fibrillation (AF) and heart failure (HF) mutually influence patients’ prognosis [1,2]. From the 1990s to the early 2000s, randomized controlled trials utilizing angiotensin-converting enzyme (ACE) inhibitors, beta-blockers, and mineralocorticoid receptor antagonists (MRAs) revealed improved outcomes of HF with a reduced ejection fraction (HFrEF). Since the late 2010s, the outcomes of randomized controlled trials using angiotensin receptor–neprilysin inhibitors and sodium–glucose cotransporter 2 (SGLT2) inhibitors have accelerated advancements in these improved prognostic effects [3,4,5,6,7,8]. Additionally, SGLT2 inhibitors have been shown to improve the prognosis of HF with a preserved ejection fraction (HFpEF), expanding the possibilities for drug therapy in HFpEF [9,10]. Despite the development of drug treatments for HF, the prognosis after the onset of HF remains very poor.

The coexistence of HF and AF is notable because of the propensity of these two conditions to mutually predispose one another [11]. AF exacerbates the prognosis of HF irrespective of cardiac function (ranging from HFrEF to HFpEF) [2]. Therefore, addressing the treatment of these shared comorbidities has become a crucial consideration. Catheter ablation (CA) for HF complicated by AF has garnered attention since the CASTLE-AF trial [12]; however, the effects of CA on hard endpoints observed in other trials have been inconsistent. Consequently, it remains unclear which subgroup within this population should be proactively selected for CA. AF is known to concomitantly occur in 20% to 35% of patients with acute decompensated HF (ADHF) [13,14]. In the present study, we hypothesized that CA is effective for patients with ADHF complicated by AF.

The primary purpose of our study was to examine the characteristics of patients with concurrent ADHF and AF, elucidating the factors that contribute to cardiovascular death and hospitalization for HF. Additionally, to assess the effectiveness of CA, we compared the event-free rates of patients who underwent the procedure with those of a matched control group.

## 2. Materials and Methods

### 2.1. Patient Selection and Exclusion Criteria

Data charts from 616 patients aged >20 years who were admitted with ADHF and presented with AF (including atrial flutter) as their cardiac rhythm were extracted consecutively from the database of the International Medical Center of Saitama Medical University in Japan, covering the period from April 2007 to March 2017.

ADHF was defined as the rapid onset or worsening of signs and symptoms caused by acute impairment of pumping function due to an organic abnormality of the heart. Here, we used the clinical scenario (CS) classification from the JCS 2017/JHFS 2017 Guideline on Diagnosis and Treatment of Acute and Chronic Heart Failure for its diagnostic classification [15,16]. This tool classifies patients with ADHF according to their pathophysiological state with reference to systolic blood pressure (CS1: initial blood pressure over 140 mmHg, CS2: 100–140 mmHg, CS3: under 100 mmHg) and provides appropriate initial management for each type of patient. Clinical information from selected patients was individually extracted from the charts, and these data were confirmed by another physician to reduce selection bias. Details of this study were published online, allowing all participants to verify their inclusion.

**Figure 1:**
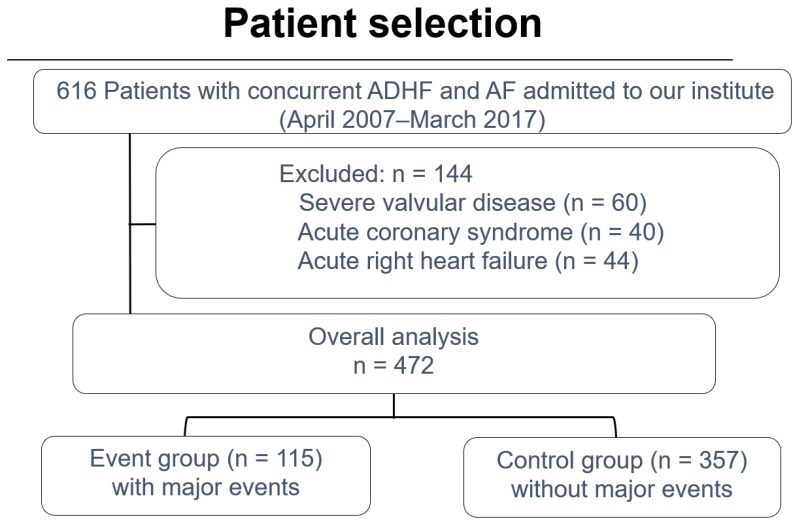
Flow diagram illustrating patient eligibility criteria. ADHF: acute decompensated heart failure; AF: atrial fibrillation.

The exclusion criteria included severe valvular disease that necessitated prioritization of other intervention therapies over ablation, CS classifications of CS4 and CS5 involving acute coronary syndrome and right heart failure, and refusal to participate in this study. In total, 472 patients were included in the analysis (Figure 1).

### 2.2. Data Collection, Follow-Up, and Clinical Outcomes

We investigated the patients’ basic characteristics, including age, sex, vital signs, CS classification, New York Heart Association [NYHA] classification, and CHADS2 score, as well as etiology, comorbidities, examination data (blood parameters and echocardiographic findings), and medications (including renin–angiotensin system inhibitors, MRAs, antiarrhythmics, beta-blockers, and diuretics). We also analyzed major event-free rates and predictors, with major events defined as a composite outcome of cardiovascular death and hospitalization for HF. Patient characteristics were compared between the major event group (event group) and other groups (control group). The follow-up period was determined at the discretion of the attending physician.

### 2.3. Procedure Setting

All ablation procedures were performed by five arrhythmia specialists certified by the Japanese Heart Rhythm Society. With the exception of amiodarone, all anti-arrhythmic drugs were discontinued for at least five half-lives before CA. Sedation was administered to maintain a Richmond Agitation Sedation Scale score of −3 to −4 during the procedure. Intravenous heparin was utilized to maintain an activated clotting time of 300 to 350 s after securing venous access. An 8-Fr 20-polar electrode catheter was inserted into the coronary sinus and/or right ventricle via the jugular, subclavian, or femoral vein. Diagnostic catheters were replaced before accessing the left atrium using the Brockenbrough method.

The CARTO^®^ Three-dimensional Mapping System (Biosense Webster, Irvine, CA, USA) was employed for AF ablation. The primary ablation strategy involved pulmonary vein isolation and tricuspid isthmus ablation. Additional procedures, such as linear ablation, complex fractionated atrial electrogram ablation, posterior wall isolation, and non-pulmonary vein foci ablation, were performed at the discretion of the physician.

A non-irrigation catheter was used from 2007 to 2012, an irrigation catheter without contact force was used from 2013 to 2014, and an irrigation catheter with contact force was used from 2015 onward.

### 2.4. Statistical Analysis

Baseline variables with normal and non-normal distributions were compared between the two groups using the *t*-test and Mann–Whitney U test, respectively. The normality of the data distribution was assessed using the Shapiro–Wilk test. The χ^2^ test was utilized for comparisons of categorical variables. The Kaplan–Meier product limit estimator was employed to estimate event-free rates. A *p*-value of <0.05 was considered statistically significant. Cox proportional hazards analysis was employed to predict the factors influencing event occurrence. Explanatory variables were chosen from significant predictors of major events identified through univariate analysis.

Propensity score matching was employed to reduce the effects of potential confounding variables. Propensity scores were estimated for each patient using logistic regression, with treatment assignment or exposure as the dependent variable and all relevant covariates as independent variables. Balance between groups after matching was assessed by calculating standardized mean differences (SMDs) for all baseline covariates. An SMD of less than 0.1 was considered indicative of adequate balance. This matching process resulted in a subset of data with balanced covariates between the treatment and control groups, allowing for a more valid comparison of outcomes.

Following matching, *t*-tests were utilized to compare continuous variables, whereas the χ^2^ test was utilized for comparisons of categorical variables in the propensity score-matched cohort. Kaplan–Meier curves and log-rank tests were performed to compare event-free rates between the two matched groups. All statistical analyses were conducted using SPSS version 21 (IBM Corp., Armonk, NY, USA).

## 3. Results

### Patients

The mean observation period was 32.8 ± 32.9 months. Table 1 shows the baseline characteristics of patients diagnosed with ADHF and concurrent AF, with 8% initially showing atrial flutter. The mean age of the patients was 69.5 ± 13.0 years, with men constituting 57% (n = 274). The mean CHADS2 score and NYHA classification were 2.2 ± 1.2 and 3.6 ± 0.6, respectively. CS1, CS2, and CS3 were present at rates of 40% (n = 191), 45% (n = 210), and 15% (n = 71), respectively. The mean left ventricular ejection fraction (LVEF) was 41.5% ± 19.3%, with HFrEF, HF with a mid-range ejection fraction (HFmrEF), and HFpEF occurring at rates of 58% (n = 281), 15% (n = 73), and 25% (n = 118), respectively. The mean left atrial diameter (LAD) was 45.2 ± 9.1 mm, the mean serum creatinine concentration was 1.5 ± 3.3 mg/dL, and the mean brain natriuretic peptide (BNP) concentration was 744.2 ± 732.8 pg/mL, all of which were higher than the reference range. Notably, chronic kidney disease (CKD) was a prevalent comorbidity (28%, n = 133).

In the comparison of the event and control groups, the former exhibited a higher mean age (72.1 ± 11.0 vs. 68.8 ± 13.4 years, *p* = 0.008) and NYHA classification (3.8 ± 0.4 vs. 3.6 ± 0.6, *p* < 0.001). The event group also demonstrated a significantly higher proportion of patients with NYHA Class 3 HF (23% vs. 12%, *p* = 0.004), along with a larger left ventricular end-systolic volume (LVESV) (83.8 ± 55.3 vs. 70.4 ± 42.5 mL, *p* = 0.031) and LAD (48.2 ± 10.2 vs. 44.8 ± 8.4 mm, *p* = 0.003). Additionally, coronary artery disease (CAD) and CKD were more prevalent in the event group than in the control group (CAD: 21% vs. 10%, *p* < 0.001; CKD: 36% vs. 26%, *p* = 0.040).

Table 2 summarizes the data collected during the follow-up period. The event group had a greater number of HF hospitalizations than the control group (1.8 ± 1.9 vs. 1.2 ± 0.6, *p* = 0.002). Furthermore, the event group exhibited a lower latest LVEF and higher latest LVESV and LAD than the control group (latest LVEF: 42.0% ± 17.8% vs. 51.9% ± 15.1%, *p* < 0.001; latest LVESV: 91.4 ± 50.2 vs. 50.7 ± 28.0 mL, *p* < 0.001; and LAD: 49.5 ± 9.1 vs. 45.5 ± 8.4 mm, *p* = 0.006). The event group showed a lower sinus rhythm (SR) maintenance rate on the last observation day than the control group (17% vs. 31%, *p* < 0.001), and a smaller proportion of patients in the event group underwent CA (9% vs. 21%, *p* = 0.003). Medication use for HF was similar between the two groups, although diuretic use was higher in the event group than in the control group (72% vs. 60%, *p* = 0.018). The rate of oral anticoagulant use was <50% in this cohort, including patients treated before direct oral anticoagulants (DOACs) became popular.

**Figure 2:**
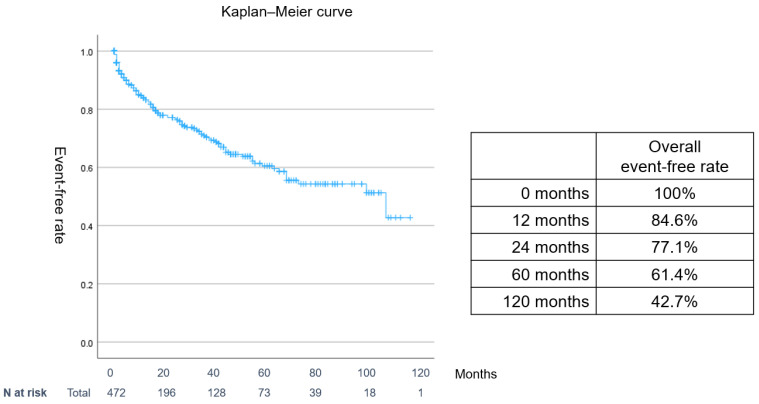
Overall event-free Kaplan–Meier curve for acute ADHF with AF. Events included cardiovascular death and hospitalization for HF. ADHF: acute decompensated heart failure; AF: atrial fibrillation; CS: clinical scenario.

Figure 2 shows the Kaplan–Meier event-free curve for all patients. The event-free rate was 84.6% at 12 months and 77.1% at 24 months. The 5-year (60-month) and 10-year (120-month) event-free rates were 61.4% and 42.7%, respectively.

**Figure 3:**
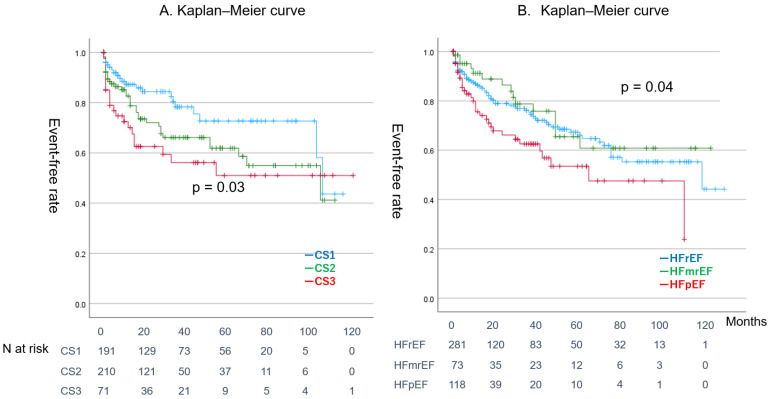
Event-free Kaplan–Meier curve categorized by different criteria. CS3 showed a significantly lower event-free rate (log-rank test: *p* = 0.01). (**A**) Blue line: CS1, green line: CS2, red line: CS3. (**B**) Blue line: HFrEF, green line: HFmrEF, red line: HFpEF.CS: clinical scenario, HFrEF: heart failure with reduced ejection fraction, HFmrEF: heart failure with mid-range ejection fraction, HFpEF: heart failure with preserved ejection fraction (**A**) Green line: CA in HFrEF, red line: non-CA in HFrEF. (**B**) Right green line: CA in non-HFrEF, orange line: non-CA in non-HFrEF.

Figure 3A,B show the event-free rates according to CS classification and LVEF status, respectively. Patients with CS3 and those with HFpEF showed a significantly lower event-free rate (CS3, log-rank test: *p* = 0.03; HFpEF, log-rank test: *p* = 0.04). However, no significant differences were observed among the HFrEF, HFmrEF, and HFpEF groups (log-rank test: *p* = 0.11).

**Figure 4:**
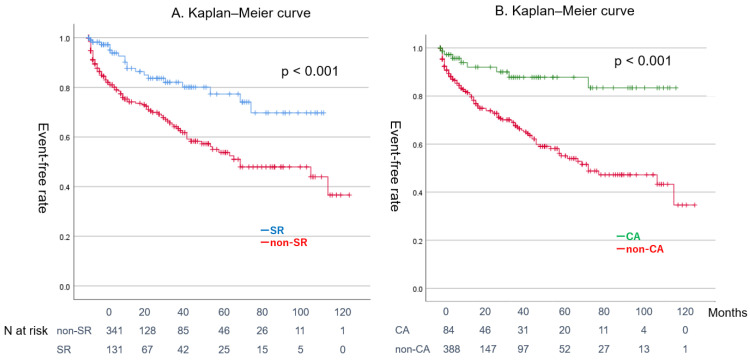
Impacts of SR maintenance and CA on event-free rate. CA: catheter ablation, SR: sinus rhythm.

**Figure 5:**
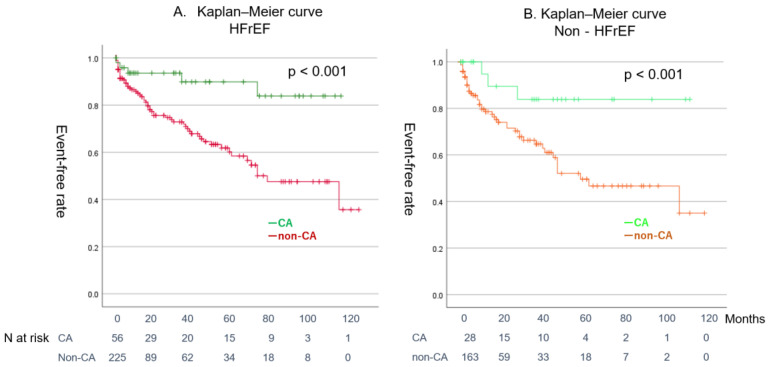
Event-free Kaplan–Meier curves for the CA and non-CA groups within the HFrEF and non-HFrEF populations.

Figure 4A shows a comparison of the event-free curves between the SR maintenance group and the non-SR group, whereas Figure 4B compares the CA group with the non-CA group. Patients in the SR maintenance group and those who underwent CA had better prognoses than their respective counterparts (log-rank test: *p* < 0.001 and *p* < 0.001, respectively). Figure 5 demonstrates a comparison of event-free rates between the CA and non-CA groups within the HFrEF and non-HFrEF populations. CA significantly reduced events in both populations (log-rank test: *p* < 0.001 and *p* < 0.001, respectively).

**Figure 6:**
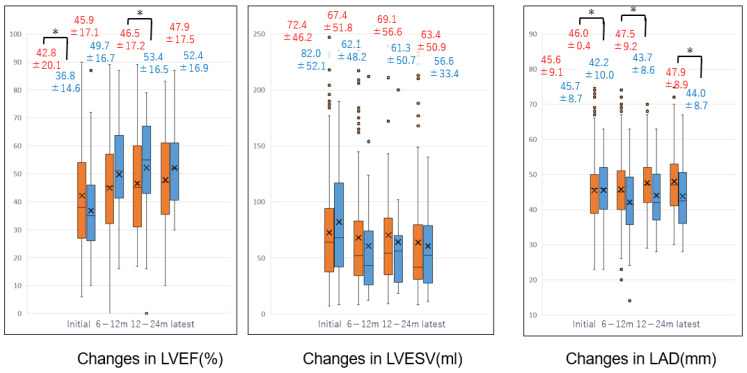
Time course of mean LVEF, LVESV, and LAD. The figure illustrates the time course of mean left ventricular ejection fraction (LVEF), left ventricular end-systolic volume (LVESV), and left atrial diameter (LAD) in the non-CA and CA groups at baseline, 6–12 months, 12–24 months, and the most recent follow-up. Orange box plots represent the non-CA group, while blue box plots represent the CA group. Numbers represent mean ± standard deviation. * indicates a significant difference between groups.

Figure 6 presents box plots illustrating changes over time in mean LVEF, mean LVESV, and LAD in the CA and non-CA groups. Echocardiographic measurements were performed during hospitalization, at 6–12 months post-discharge, 12–24 months, and at the most recent follow-up. Box plots for the CA group are shown in blue, while those for the non-CA group are in orange. At baseline, LVEF was significantly lower in the CA group but surpassed that of the non-CA group at 12–24 months (baseline LVEF: CA group vs. non-CA group = 36.8 ± 14.6% vs. 42.8 ± 20.1%, *p* = 0.003). Although LVESV was smaller in the CA group compared to the non-CA group at 6–12 months and later, the difference was not statistically significant. The most pronounced change was observed in LAD. While initial LAD values were similar between groups, LAD was significantly smaller in the CA group at 6–12 months, 12–24 months, and at the most recent follow-up (baseline LAD: CA group vs. non-CA group = 45.7 ± 8.7 mm vs. 45.6 ± 9.1 mm, *p* = 1.0; 6–12 months: 42.2 ± 10.0 mm vs. 46.0 ± 10.4 mm, *p* = 0.008; 12–24 months: 43.7 ± 8.6 mm vs. 47.5 ± 9.2 mm, *p* = 0.02; latest: 44.0 ± 8.7 mm vs. 47.9 ± 8.9 mm, *p* = 0.02).

Table 3 presents the hazard ratios for major events obtained from the univariate and multivariate analyses. In the univariate analysis, baseline categories, including NYHA Class 3 and 4 HF, CS3, a high LVESV and LAD, as well as comorbidities such as CAD and CKD, emerged as significant predictors of events. Additionally, factors in follow-up categories, including multiple hospitalizations, CA, SR maintenance, and diuretic use, were identified as significant predictors. Notably, multiple hospitalizations, CA, and diuretic use remained significant predictors in the multivariate analysis.

Table 4 presents the characteristics of patients in the matched CA and non-CA groups after pre- and post-propensity score matching. Both groups were statistically equivalent in terms of age, sex, NYHA classification, CHADS2 score, LVEF, and LAD. Before matching, the CA group was significantly younger than the non-CA group, had lower CHADS2 scores and NYHA classifications, lower BNP levels, and a lower LVEF (see Supplementary Table S1 for more details). We selected age, sex, CHADS2 score, NYHA classification, LVEF, and LAD for matching, as previous studies have identified these factors as significant predictors of prognosis in AF and HF [17,18,19,20]. After matching, significant improvements in LVEF, LAD, and BNP concentration were observed in the CA group. Among the patients in the CA group, 35% (n = 17) experienced recurrence, with 31% (n = 12) undergoing a second ablation procedure. The SR maintenance rate was higher in the matched CA group than in the matched non-CA group (73% vs. 33%, *p* < 0.001). CA significantly improved LVEF and significantly decreased LAD and serum BNP concentration.

**Figure 7:**
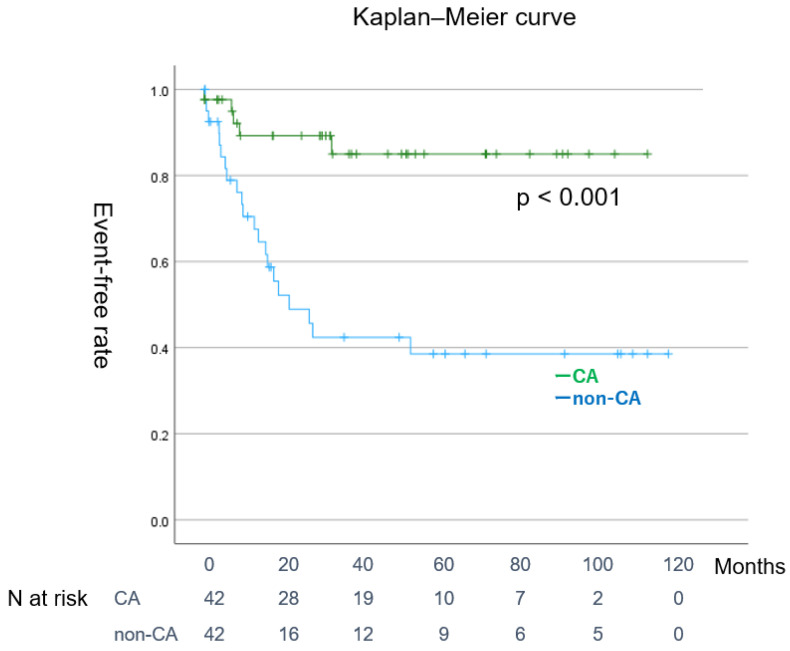
(Representative figure). Event-free Kaplan–Meier curve for the CA and non-CA groups after matching. CA: catheter ablation.

Figure 7 shows that the event-free rate of the matched CA group was significantly lower than that of the matched non-CA group (log-rank test: *p* < 0.001).

## 4. Discussion

### 4.1. Major Findings

This retrospective study yielded several pivotal insights. First, patients who experienced major events (defined as cardiovascular death and hospitalization for HF) during the observation period tended to be older and predominantly male and have higher CHADS2 scores. Although there was no significant difference in cardiac function between the event group and the control group, the LVESV and LAD were larger in the event group than in the control group. Additionally, the event group had a higher prevalence of CAD and CKD as comorbidities.

Second, the prognosis for patients with ADHF presenting with AF upon hospitalization was notably poor, with a 5-year event-free rate of 61.4% and a 10-year event-free rate of 42.7%. While the event rate was elevated in patients with CS3, no differences were observed in the event rates across functional classifications such as HFrEF, HFmrEF, and HFpEF. Although the event rate was elevated in patients with CS3 in the CS category, HFpEF had the highest event rate among the functional classifications, such as HFrEF, HFmrEF, and HFpEF.

Third, the event group demonstrated high rates of hospitalization for HF, diminished cardiac function, and unfavorable remodeling of both the left ventricle and left atrium during the follow-up period. In contrast, the control group maintained a significantly higher SR maintenance rate and a superior CA rate compared with the event group. Although drug therapy was comparable between the two groups, the use of diuretics was significantly more prevalent in the event group.

Finally, maintaining SR and undergoing CA were associated with improved cardiac function, a lower BNP concentration, and a more favorable prognosis. CA also inhibited cardiac remodeling, particularly in the left atrial chamber. These effects persisted even after adjusting for the patients’ background characteristics through propensity score matching.

### 4.2. Relationship Between AF and HF Complexity of Atrial Fibrillation and Heart Failure

Since the 1990s, significant advancements in medical therapy for HF, such as the adaptation of ACE inhibitors, beta-blockers, and MRAs, have notably enhanced the prognosis of HFrEF [3,4,5]. Attention has recently turned toward the “fantastic four” treatment strategy, which incorporates angiotensin receptor–neprilysin inhibitors (as ACE inhibitor replacements) and SGLT2 inhibitors [15]. However, attaining an overall improvement in the prognosis of HF has been challenging, partly because of the high prevalence of HFpEF, which comprises nearly half of all HF cases, as well as limited established methods for prognosis enhancement and high rates of acute mortality and readmission for ADHF [16,17].

The incidence of AF is higher in patients with HF than in those without HF, and patients with AF are predisposed to developing HF over time [18]. Additionally, many studies have demonstrated a high rate of SR maintenance after CA, with similar outcomes observed in patients with concurrent HF and AF [11,19].

Although the sample size was small, our previous study showed that AF ablation for HFrEF resulted in the recovery of left ventricular and left atrial function, a finding supported by systematic reviews [20,21]. However, the impact on hard endpoints, such as hospitalization for HF and cardiovascular death, remained unclear until the CASTLE-AF trial in 2018, which demonstrated the positive effects of CA for patients with AF who had an implantable cardioverter-defibrillator (ICD) or cardiac resynchronization therapy-defibrillator (CRTD) [12].

Despite some positive outcomes, the efficacy of AF ablation was attenuated in the CASTLE-AF trial among groups with more severe HF, such as those with an LVEF of <25% and NYHA Class 3 HF. The next challenge lies in determining which subtypes of HF benefit most from AF ablation [22,23].

Despite recent advances in HF pharmacotherapy, particularly the introduction of the so-called “Fantastic Four”, the overall prognosis for HF has not significantly improved over the past decades [21]. One major contributing factor is the persistently poor prognosis associated with ADHF and HF complicated by AF [22,23]. The effectiveness of conventional drug therapy in HF patients with AF remains limited, and the optimal heart rate target for rate control strategies differs from that in sinus rhythm [24]. Moreover, the efficacy of beta-blockers, which play a pivotal role in the management of both HF and heart rate control, is reduced in this population, making it challenging to maintain sinus rhythm through pharmacological means alone [25].

There is now robust evidence supporting the use of CA in HF with AF, spanning from HFrEF to HFpEF, as demonstrated by trials such as the CASTLE-AF trial [12,26,27,28,29]. In the 2024 EHRA/HRS/APHRS consensus statement, CA for HF with AF has been upgraded to a Class I recommendation [30]. However, ablation may not be suitable for all HF patients with AF, and further research is required to identify subgroups that may benefit most from this intervention. We focused on ADHF, which continues to carry a poor prognosis. Approximately 20% to 35% of patients with ADHF also have AF, and this combination is associated with poorer clinical outcomes. The CASTLE-AF population is relatively similar to that of our study in terms of age and gender, but there has been no analysis of patients with or without a history of ADHF [12]. In addition, HFmrEF and HFpEF were not included. In this respect, our study provides a new perspective. One study showed that among patients admitted to the hospital with acute HF, current AF, or a history of AF was associated with less improvement in dyspnea and higher morbidity and mortality rates at 30 days [31]. However, the prognostic factors within this subgroup remain unclear.

Although many HF studies for HFpEF include patients with AF (32–57%), advancing HF treatment in this population remains crucial. While angiotensin receptor–neprilysin inhibitors (ARNIs) demonstrated efficacy in HFpEF, the difference did not reach statistical significance. SGLT2 inhibitors were the first drugs to show clear efficacy in HFpEF. More recently, glucagon-like peptide-1 (GLP-1) receptor agonists have been shown to improve outcomes in both HFrEF and HFpEF, particularly in patients with obesity [32,33]. GLP-1 receptor agonists have also demonstrated potential in reducing recurrence after CA, suggesting their possible effectiveness in ADHF patients with AF. However, these agents were not utilized during the observation period of this study, and addressing this issue is the next challenge [34]. According to the analysis in the OPTIMIZE-HF trial (in which 31% of patients had AF), predictors of early mortality after discharge from hospitalization for ADHF included higher age (1.22 hazard ratio for each 10-year increase in age), higher serum creatinine concentration (1.32 hazard ratio for each 4-mg/dL increase in creatinine), reactive airway disease, liver disease, lower systolic blood pressure, lower serum sodium concentration, lower admission weight, and depression [35]. In our study population of patients with AF, we identified older age and CKD as poor prognostic factors, with a significantly higher CS (CS3). However, reactive airway disease, liver disease, and depression were not investigated in our study.

The relationship between acute-phase echocardiographic parameters and prognosis in patients with concurrent ADHF and AF has not been extensively studied. Kim et al. [36]. reported that left ventricular end-diastolic diameter was a prognostic factor for ischemic heart failure in a Korean registry in which 24.4% of patients had AF. Notably, in the present study, the LAD and LVESV upon admission were greater in the event group than in the control group. However, elucidating the implications of this higher LAD and LVESV warrants further investigation into myocardial damage and cardiac reverse remodeling.

### 4.3. Clinical Outcomes of Patients with Concurrent ADHF and AF During Follow-Up

In the present study, the frequency of hospitalization and the utilization of diuretics were higher in the event group than in the control group. Multiple hospitalizations and diuretic use were also highly significant predictors of major events in the multivariate analysis. The number of hospitalizations for HF has been identified as a poor prognostic factor in patients with HF [37]. Furthermore, persistent congestion following initial treatment for ADHF and prolonged diuretic use in patients with chronic HF have been associated with a worse prognosis [38,39]. Our findings align with these observations, particularly among patients with concurrent AF and ADHF.

The CA group and the group that maintained SR at the final follow-up demonstrated lower rates of major events than their respective counterparts. A subanalysis of the well-known AFFIRM study, which showed no difference in mortality between the rhythm control group and the rate control group among patients with AF, indicated that the prognosis for patients with SR maintenance was favorable [40].

However, accurately assessing SR maintenance may be challenging because recurrence often develops without accompanying symptoms. Notably, the subanalysis of the CASTLE-AF trial, which allowed for the precise evaluation of recurrence because of the inclusion of patients with ICD and CRTD, demonstrated that a <50% decrease in the AF burden improved prognosis [41]. Although AF burden assessment was not feasible in our study, our results advocate for striving toward SR maintenance in patients with combined ADHF and AF.

The LAD and LVESV remained larger in the event group than in the control group throughout the follow-up period. In the context of HF treatment (both pharmacological and non-pharmacological), left ventricular reverse remodeling serves as a crucial prognostic indicator and may lead to left atrial remodeling. Previous studies have shown that AF ablation induces both left ventricular and left atrial reverse remodeling [42,43]. Our study supports this trend, highlighting that AF ablation particularly facilitates left atrial reverse remodeling. Direct intervention on the left atrium may enhance the HF-improving effects of pharmacological therapy, acting as a potential booster. However, further investigation is necessary to clarify this mechanism. At the very least, our findings underscore the significance of reverse remodeling in both the left atrium and left ventricle as prognostic markers in patients with ADHF and AF.

### 4.4. Effect of CA on Patients with Combined ADHF and AF

When AF and HF coexist, the prognosis is generally worse when HF precedes AF compared to cases in which AF precedes HF [44]. However, in many cases of ADHF, it remains unclear which condition arises first, and there is a lack of prognostic studies specifically focused on this population.

As anticipated, patients in our cohort who had both ADHF and AF exhibited an exceedingly poor prognosis. Variations in LVEF had a minimal impact on prognosis, with CS3 indicating a notably poorer outcome. Komuro et al. [45] noted a worse prognosis associated with CS3 in patients with ADHF in Japan, with approximately 53% of the study population presenting with AF. In the WET-HF Registry, which is a registry of ADHF that includes approximately 53% of AF patients, CS3 has been reported to be associated with poor prognosis across all EF categories, although this association is attenuated in CS3 of HFpEF [45]. Considering that cases of severe valvular disease were excluded from our subjects, unlike in WET-HF, these findings did not deviate significantly from the results of the present study.

Since the CASTLE-AF trial [12], the scope of AF ablation in patients with concomitant HF and AF has expanded. However, the specific impact of AF ablation on the combination of ADHF and AF remains largely unexplored.

In the present study, CA was found to significantly improve the prognosis in patients with ADHF, and this effect persisted even after propensity matching to align the patients’ backgrounds. Furthermore, CA induced reverse remodeling of the left ventricle and left atrium, leading to a marked decrease in BNP concentration. Two reports have documented the efficacy of acute-phase AF ablation for patients with ADHF exhibiting unstable hemodynamics [46,47].

Sakamoto et al. [48] demonstrated the effectiveness of CA within 90 days for patients with combined ADHF and AF in a large-scale registry in Japan. However, their study did not include a matched control group.

The findings of our study suggest that the favorable outcomes of CA for combined ADHF and AF persist even after matching. Thus, CA should be actively considered for patients presenting with concurrent ADHF and AF. It is important to note that in the pre-matching comparison, the non-CA group was older, had higher CHADS2 and NYHA scores, and better LVEF than the CA group. Unfortunately, high-risk cases with relatively preserved cardiac function may not be favorably chosen as candidates for ablation.

### 4.5. Clinical Implication

In this single-center study, CA performed during hospitalization for ADHF in patients with concomitant AF significantly improved prognosis, and this benefit was maintained even after matching. These findings underscore the potential clinical utility of CA in these patients.

### 4.6. Study Limitations

This study is subject to the limitations inherent in single-center, retrospective investigations. Follow-up for each patient was conducted at the discretion of the attending physician, without a standardized protocol, potentially introducing bias in decisions regarding discontinuation of follow-up. To emphasize the effects of AF and CA, patients with severe valvular disease, particularly mitral regurgitation, were excluded. As a result, this study did not encompass all patients with heart failure and concomitant atrial fibrillation, and the possibility of selection bias cannot be ruled out. Furthermore, the number of cases was insufficient to perform a three-group comparison in CS classification or classification based on EF, which may have led to statistical underpowering. Propensity score matching was employed to address apparent differences in patient backgrounds between the CA and non-CA groups. However, the number of parameters available for matching was limited, and the risk of arbitrariness in their selection cannot be entirely eliminated. Moreover, the observational period of this study began in 2007 and spanned 11 years, preceding the widespread use of DOACs. Furthermore, current clinical practices, including notable outcomes associated with HF medications, such as the “fantastic four” strategy and SGLT2 inhibitors for HFpEF, may differ from those observed in cases predating the adoption of DOACs and these specific therapeutic interventions. Additionally, novel CA techniques, such as high-density mapping systems and pulse field ablation, have advanced, becoming more precise and safer. Despite the technical limitations relative to current standards, we believe the benefits of CA observed in this study are still clinically significant.

## 5. Conclusions

Patients presenting with AF at the onset of ADHF exhibited a poor prognosis. Although CA showed promise in improving the prognosis for these patients, further research is warranted to identify high-priority candidates for CA.

## Figures and Tables

**Table 1:** Patients’ characteristics.

Total Number	Total (n = 472)	Event Group (n = 115)	Control Group (n = 357)	*p*-Value	
Male	274 (57%)	74 (64%)	200 (56%)	0.116	NS
Age	69.5 ± 13.0	72.1 ± 11.0	68.8 ± 13.4	0.008	
CHADS2 score	2.2 ± 1.2	2.4 ± 1.3	2.2 ± 1.2	0.073	NS
NYHA classification	3.6 ± 0.6	3.8± 0.4	3.6 ± 0.6	<0.001	
BMI	21.9 ± 3.5	21.2 ± 3.5	22.3 ± 4.6	0.221	NS
Systolic BP(mmHg)	134.1 ± 36.0	132.0 ± 37.3	135.6 ± 36.6	0.565	NS
HR (bpm)	111.9 ± 36.8	105.1± 37.2	110.3 ± 35.2	0.364	NS
Categories					
CS1	191 (40%)	44 (38%)	147 (30%)	0.580	NS
CS2	210 (44%)	43 (37%)	167 (47%)	0.078	NS
CS3	71 (15%)	28 (24%)	43 (12%)	0.001	
HFrEF	281 (58%)	66 (64%)	215 (65%)	0.590	NS
HFmrEF	73 (15%)	15 (15%)	58 (18%)	0.409	NS
HFpEF	118 (24%)	34 (30%)	84 (24%)	0.194	NS
Examinations					
LVEF (%)	41.5 ± 19.3	42.9 ± 21.8	41.4± 18.5	0.531	NS
LVESV (mL)	73.6 ± 48.4	83.8 ± 55.3	70.4 ± 42.5	0.031	
LAD (mm)	45.2 ± 9.1	48.2 ± 10.2	44.8 ± 8.4	0.003	
Cr (mg/dL)	1.5 ± 3.3	1.4 ± 1.2	1.6 ± 3.8	0.532	NS
Hb (g/dL)	12.9 ± 2.5	12.4 ± 2.3	14.2 ± 21.9	0.168	NS
BNP (pg/mL)	744.2 ± 732.8	758.7 ± 703.1	748.4 ± 756.3	0.905	NS
Comorbidities					
PAF	110 (23%)	33 (33%)	77 (22%)	0.116	NS
DM	83 (18%)	27 (23%)	56 (16%)	0.056	NS
HT	199 (42%)	48 (42%)	151 (42%)	0.916	NS
CVD	67 (14%)	21 (18%)	46 (13%)	0.151	NS
CAD	58 (12%)	24 (21%)	34 (10%)	<0.001	
CKD	133 (28%)	41 (36%)	92 (26%)	0.040	

Data are presented as number (percentage) of patients or mean ± standard deviation. NYHA: New York Heart Association, BMI: body mass index, BP: blood pressure, HR: heart rate, CS: clinical scenario, HFrEF: heart failure with reduced ejection fraction, HFmrEF: heart failure with mid-range ejection fraction, HFpEF: heart failure with preserved ejection fraction, LVEF: left ventricular ejection fraction, LVESV: left ventricular end-systolic volume, LAD: left atrial diameter, Cr: serum creatinine, Hb: hemoglobin, BNP: serum brain natriuretic peptide, PAF: paroxysmal atrial fibrillation, DM: diabetes mellitus, HT: hypertension, CVD: cerebrovascular disease, CAD: coronary artery disease, CKD: chronic kidney disease, NS: not significant.

**Table 2:** Data analyzed during the study period.

	Total		Event Group		Control Group		*p*-Value	
Total Number	(n = 472)		(n = 115)		(n = 357)			
Single HFH	311 (57%)		65 (57%)		246 (69%)		0.015	
Multiple HFH	161 (34%)		50 (43%)		111 (31%)		0.015	
Total N of HFH	1.3 ± 1.1		1.8 ± 1.9		1.2 ± 0.6		0.002	
Latest LVEF(%)	48.7 ± 16.7		42.0 ± 17.8		51.9 ± 15.1		<0.001	
Latest LVESV(mL)	63.8 ± 50.2		91.4 ± 50.2		50.7 ± 28.0		<0.001	
Latest LAD(mm)	46.8 ± 9.1		49.5 ± 9.1		45.5 ± 8.4		0.006	
SR maintenance	131 (28%)		20 (17%)		111 (31%)		<0.001	
Procedures								
CA	84 (17%)		10 (9%)		74 (21%)		0.003	
before contact force	70 (14%)		8 (7%)		62 (18%)		0.373	NS
after contact force	14 (3%)		2 (2%)		12 (3%)		0.373	NS
Pacemaker	10 (2%)		3 (3%)		7 (2%)		0.675	NS
ICD	21 (4%)		8 (7%)		13 (4%)		0.134	NS
CRT	15 (3%)		7 (6%)		8 (2%)		0.041	
other intervention	16 (3%)		7 (6%)		9 (3%)		0.066	NS
Medications								
Anti-platelet	131 (28%)		33 (29%)		98 (27%)		0.795	NS
OAC	224 (47%)	[DOAC16%]	52 (45%)	[DOAC13%]	172 (48%)	[DOAC17%]	0.580	NS
BB	224 (47%)		51 (45%)		173 (48%)		0.443	NS
RAAS antagonist	249 (52%)		64 (56%)		185 (52%)		0.474	NS
AAD	156 (32%)		36 (31%)		120 (34%)		0.980	NS
Amiodarone	98 (21%)		26 (23%)		72 (20%)		0.575	NS
Diuretics	297 (62%)		83 (72%)		214 (60%)		0.018	

Data are presented as number (percentage) of patients or as mean ± standard deviation. HFH: heart failure hospitalization, LVEF: left ventricular ejection fraction, LVESV: left ventricular end-systolic volume, LAD: left atrial diameter, SR: sinus rhythm, CA: catheter ablation, ICD: implantable cardioverter-defibrillator, CRT: cardiac resynchronization therapy, OAC: oral anticoagulants, DOAC: direct oral anticoagulants, BB: beta-blockers, RAAS: renin–angiotensin–aldosterone system, AAD: anti-arrhythmic drugs, NS: not significant.

**Table 3:** Event hazard ratios from the univariate and multivariate analyses.

	Univariate Analysys			Multivariate Analysys		
Categories	HR	CI	*p*-Value	HR	CI	*p*-Value
Baseline						
Age > 74 y	1.20	(0.78–1.83)	0.403			
NYHA classification 3/4	1.53	(0.97–2.43)	0.070			
CS 3	2.20	(1.28–3.75)	0.004			
LVEF < 40%	0.75	(0.50–1.14)	0.180			
LVESV > 84 mL	1.85	(1.25–2.80)	0.003			
LAD > 44 mm	1.74	(1.13–2.69)	0.012			
CAD	2.53	(1.42–4.53)	0.002			
CKD	1.66	(1.04–2.67)	0.036			
Follow up						
multiple hospitalizatioin	4.53	(2.60–7.91)	<0.001	2.26	(1.41–3.61)	<0.001
CA	0.36	(0.18–0.71)	0.004	0.41	(0.21–0.82)	0.012
SR maintenance	0.46	(0.27–0.78)	0.004			
Deuretics	3.02	(1.69–5.41)	<0.001	2.20	(1.27–3.80)	0.005

HR: hazard ratio, CI: 95% confidence interval, NYHA: New York Heart Association, CS: clinical scenario, LVEF: left ventricular ejection fraction, LVESV: left ventricular end-systolic volume, LAD: left atrial diameter, CAD: coronary artery disease, CKD: chronic kidney disease, CA: catheter ablation, SR: sinus rhythm.

**Table 4:** Comparison of patient characteristics between CA group and non-CA group pre- and post-matching.

Pre Matching	CA Group (n = 84)	Non-CA Group (n = 388)	*p*-Value	
Age	61.5 ± 13.0	66.2 ± 11.2	<0.001	
Male	56 (67%)	218 (56%)	0.440	NS
CHADS2 score	1.6 ± 1.0	2.3 ± 1.2	<0.001	
NYHA	3.2 ± 0.6	3.7 ± 0.5	<0.001	
preLVEF (%)	36.8 ± 14.6	42.8 ± 20.1	0.003	
latestLVEF (%)	50.9 ± 15.4	47.7 ± 17.2	0.225	NS
preLAD (mm)	45.7 ± 8.7	45.6 ± 9.1	0.951	NS
latestLAD (mm)	43.8 ± 9.3	47.9 ± 8.9	0.009	
preBNP (pg/mL)	567.5 ± 511.2	787.7 ± 777.5	0.005	
latestBNP (pg/mL)	71.6 ± 87.0	1041.0 ± 4870.1	<0.001	
Recurence after 1stCA	48 (57%)	NA	NA	
Second ablation	27 (32%)	NA	NA	
SR maintenance	54 (64%)	77 (20%)	<0.001	
Post Matching	(n = 42)	(n = 42)		
Age	60.3 ± 11.3	66.2 ± 11.2	0.110	NS
Male	30 (62%)	34 (70%)	0.440	NS
CHADS2 score	1.6 ± 0.8	1.5 ± 1.0	0.790	NS
NYHA	2.4 ± 1.0	2.8 ± 0.8	0.340	NS
preLVEF (%)	35.6 ± 15.9	40.0 ± 17.0	0.470	NS
latestLVEF (%)	59.1 ± 13.8	43.9 ± 18.5	<0.001	
preLAD (mm)	44.3 ± 9.2	44.2 ± 11.2	0.230	NS
latestLAD (mm)	42.1 ± 13.8	49.1 ± 13.5	0.047	
preBNP (pg/mL)	463.1 ± 407.4	713.2 ± 722.6	0.070	NS
latestBNP (pg/mL)	71.6 ± 87.0	671.9 ± 806.2	<0.001	
Recurence after 1st CA	17 (40%)	NA	NA	
Second ablation	13 (31%)	NA	NA	
SR maintenance	31 (73%)	14 (33%)	<0.001	

Age, sex, CHADS2 score, NYHA classification, LVEF, and LAD have been identified in a previous study as poor prognostic factors for HF and AF. After matching, the analysis compared 42 matched cases. Data are presented as the number (percentage) of patients or as mean ± standard deviation. CA: catheter ablation, NYHA: New York Heart Association, LVEF: left ventricular ejection fraction, LAD: left atrial diameter, BNP: serum brain natriuretic peptide, SR: sinus rhythm, NA: not applicable, NS: not significant.

## Data Availability

The data underlying this article will be shared upon reasonable request to the corresponding author.

## Supplementary Materials

The following supporting information can be downloaded at https://www.mdpi.com/article/10.3390/jcm14020629/s1: Table S1: Comparison of patient characteristics between the ablation group and the non-ablation group.
