# Supplementary figures and images for: Clinical Outcomes of Catheter Ablation for Atrial Fibrillation in Patients with Acute Decompensated Heart Failure
Source: J Clin Med. 2025 Jan 19;14(2):629. doi: 10.3390/jcm14020629 (PMC11765677; doi:10.3390/jcm14020629)

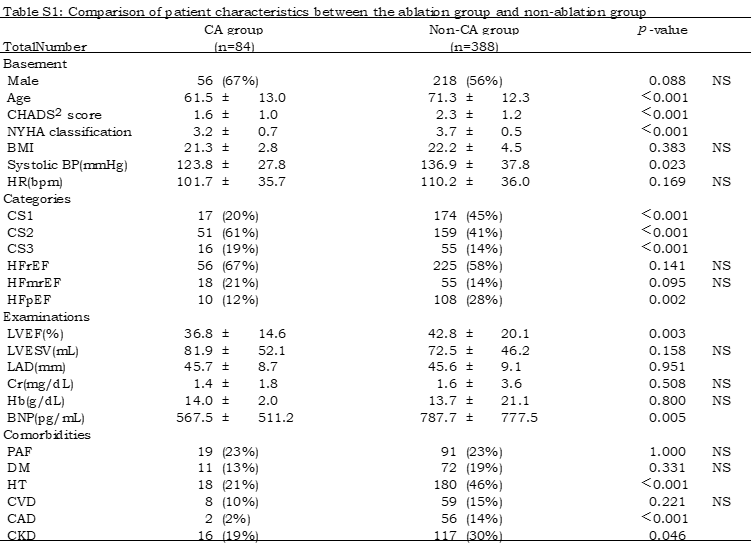

Supplement: Supplementary file 1 [file jcm-14-00629-s001.zip › Table S1.png]
